# Three Growth Spurts in Global Physical Activity Policies between 2000 and 2019: A Policy Document Analysis

**DOI:** 10.3390/ijerph19073819

**Published:** 2022-03-23

**Authors:** Trish Muzenda, Maylene Shung-King, Estelle Victoria Lambert, Anna Brugulat Panés, Amy Weimann, Nicole McCreedy, Lambed Tatah, Clarisse Mapa-Tassou, Ishtar Govia, Vincent Were, Tolu Oni

**Affiliations:** 1 Research Initiative for Cities Health and Equity (RICHE), Division of Public Health Medicine, School of Public Health and Family Medicine, University of Cape Town, Cape Town 7925, South Africa; amy.weimann@uct.ac.za (A.W.); tolullah.oni@mrc-epid.cam.ac.uk (T.O.); 2 Global Diet and Physical Activity Research, MRC Epidemiology Unit, University of Cambridge, Cambridge CB2 0QQ, UK; anna.brugulat@mrc-epid.cam.ac.uk (A.B.P.); lambed.tatah@mrc-epid.cam.ac.uk (L.T.); 3 Health Policy and Systems Division, School of Public Health and Family Medicine, University of Cape Town, Cape Town 7925, South Africa; maylene.shungking@uct.ac.za (M.S.-K.); mccnic003@myuct.ac.za (N.M.); 4 Research Centre for Health through Physical Activity, Lifestyle and Sport (HPALS), FIMS International Collaborating Centre of Sports Medicine, Department of Human Biology, Division of Physiological Sciences, Faculty of Health Sciences, University of Cape Town, Cape Town 7725, South Africa; vicki.lambert@uct.ac.za; 5 African Centre for Cities, University of Cape Town, Cape Town 7701, South Africa; 6 Health of Populations in Transition Research Group (HoPiT), University of Yaoundé I, Yaoundé P.O. Box 8046, Cameroon; mapatassou@yahoo.fr; 7 Department of Public Health, Faculty of Medicine and Pharmaceutical Sciences, University of Dschang, Dschang P.O. Box 96, Cameroon; 8 Caribbean Institute for Health Research, Mona Campus, The University of the West Indies, Kingston 7, Jamaica; ishtargovia@gmail.com; 9 Center for Global Health Research, Kenya Medical Research Institute (KEMRI), P.O. Box 1578, Kisumu 40100, Kenya; vincentwere@gmail.com

**Keywords:** physical activity, policy, intersectoral action, noncommunicable disease(s)

## Abstract

Non-communicable diseases (NCDs) contribute significantly to global mortality and are of particular concern in growing urban populations of low- and-middle income countries (LMICs). Physical inactivity is a key NCD determinant and requires urgent addressing. Laudable global and regional efforts to promote physical activity are being made, but the links between physical activity (PA), NCD reduction, and integrated intersectoral approaches to reducing obesogenic environments are not consistently made. This study applied a document analysis approach to global PA and NCD policies to better understand the current global policy environment and how this may facilitate integrated PA promotion. A total of 34 global policies related to PA, from different sectors, were analyzed. PA policy in mitigation of NCDs has evolved exponentially, with a progression towards addressing structural determinants alongside individual behavior change. The global PA agenda is primarily driven by the World Health Organization. Intersectoral collaboration is importantly regarded, but the contributions of other sectors, outside of health, education, transport, and urban planning, are less clear. Improving PA among key sub-populations—women, girls, and adolescents—requires greater policy consideration. It is imperative for PA-relevant sectors at all levels to recognize the links with NCDs and work towards integrated policy and practice in mitigation of the rising NCD pandemic.

## 1. Introduction

Physical inactivity is a key global health concern [1]. Since 2001, it has been estimated that over a quarter of the global adult population is physical inactive [2,3] as they do not meet the recommended physical activity (PA) levels of at least 150 min of moderate to vigorous intensity PA per week [4]. Physical inactivity is even higher amongst adolescents [4]. In 2016, a staggering 81% of adolescents aged 11–17 years did not engage in the recommended 60 min of moderate to vigorous physical intensity per day [5]. Moreover, patterns of physical inactivity vary according to country income groups, with high-income countries (HIC) reporting prevalence rates of physical inactivity that are twice as high as their low- and middle-income country (LMIC) counterparts [4].

While physical inactivity is a global pandemic in its own right [1], its relevance in public health is heightened by its association with non-communicable disease (NCD) occurrence [6]. Epidemiological evidence has identified physical inactivity and unhealthy diets as important risk factors for NCD precursor conditions—including overweight and hypertension—as well as a direct risk factor for key NCDs—chronic respiratory diseases, cancer, cardiovascular disease, and diabetes [6,7,8]. The profound implications of physical inactivity for various NCDs render it as an important entry point for NCD prevention and treatment [9,10]. Moreover, being a behavioral risk factor influenced by structural determinants makes it amenable for intervention at the micro, meso, and macro levels [9,10], with micro-level interventions focused on individual behavior; meso-level interventions focused on organizational and community behavior/strategies; and macro-level interventions centered on addressing structural determinants through policy and related social, economic, commercial, and environmental strategies, amongst others.

The global extent of physical inactivity and its implications has received much attention. Over the past decades, various international organizations and associations have held discussions on the causes and current state of physical inactivity, as well as key strategies for mitigation, many of which require upstream policy actions outside the health sector [11,12,13]. These discussions have reflected the recognition that determinants of PA are multifaceted and include socio-cultural, economic, built environment, and governance factors [14,15,16]. As such, strategies for reducing global physical inactivity are multifaceted and require sustained intersectoral collaboration and action from a diverse group of actors and sectors.

As the global discourse on PA and health becomes more nuanced, there has been an observable increase in PA considerations within global policies. This is evidence in policies from the leading global health agency—the World Health Organization (WHO) [11,17,18], international societies focused on PA—such as the International Society for Physical Activity and Health (ISPAH) [13], and developmental organizations—such as the United Nations Human Settlements Programme (UN-Habitat) [19]. The burgeoning of such policies is a testament to global PA advocacy and positive steps towards PA promotion. As such, there is a need to collectively dissect the contents of existing policies to understand the global organizational position, recommendations, and commitments towards PA, in relation to health, and, more recently, development. To our knowledge, there has not been an academic review of current global physical activity policies to illuminate the gap in our understanding of how global physical activity policies promote PA and accompanying policy implications.

Against this context, we conducted a desk-based document analysis of global policy documents that address PA, published between 2000 and 2019. The objectives of the study were as follows:

I. Explore the PA policy development trajectory and evolution across these two decades.
II. Conduct a policy content analysis [20] to elucidate the current landscape of global policies for promotion of increased PA.
III. Explore the extent to which policies targeted children and adolescents, given the importance of promoting PA as early as possible in the life course [21], as well as whether and how gender was addressed, given the gendered nature of PA opportunities and participation [22,23], especially in LMIC contexts.

The current study was conducted as part of the Global Diet and Activity Research (GDAR) network portfolio of projects. The GDAR network seeks to support the prevention of NCDs in LMICs, with a specific focus on Kenya, Cameroon, South Africa, and Jamaica, by addressing the knowledge gaps on upstream factors contributing to the NCD epidemic in these contexts [24]. This study is part of a GDAR work package that explored the multilevel policy landscape for intersectoral and multisectoral policies that promote healthy food environments (through reduced sugar and salt intake) and healthy placemaking (to increase PA) to prevent NCDs globally and in LMIC settings. The findings presented in this paper focus specifically on global policies that influence PA behavior.

## 2. Materials and Methods

### 2.1. Document Search

For the purpose of this research, global PA policies included all policy statements, declarations, policy guidelines, and policy proposals published from 2000 to 2019 [25]. Between June and July 2019, N.M. and A.W. systematically searched the websites and databases of international organizations from the health, transport, sports and recreation, education, youth affairs, and urban development sectors. A hand search for relevant documents was conducted in the following organizational websites: World Health Organization (WHO), United Nations Global Urban Observatory, United Nations Human Settlements Programme (UN-Habitat), Centers for Disease Control and Prevention (CDC), Global Health Data Exchange (GHDx), The United Nations Children’s Fund (UNICEF), World Bank Group (WBG), United Nations Development Programme (UNDP), United Nations Sustainable Development Knowledge Platform, United Nations Digital Library, NCD Risk Factor Collaboration (NCD-RisC), United Nations High Commissioner for Refugees (UNHCR), Global Youth Advisory Council (GRYC), United Nations General Assembly, and the United Nations Population Division. These sectors, and organizations were selected on the basis of their relevance to PA promotion, NCD prevention, and health. The following search terms were used: “physical activity”, “physical inactivity”, “exercise”, “non-communicable disease(s)”, “sedentary”, “physical education”, “exercise, sport”, “walking”, “cycling”, and “public transport”.

Within each website, policy documents were searched for under the legislation, publications, resources, or conferences sections. Where applicable, searches were conducted for specific topics (PA, urban health, and transport) or using the term “physical activity” to retrieve relevant documents. These documents were scanned to ascertain whether they made reference PA.

### 2.2. Document Screening

**Figure 1:**
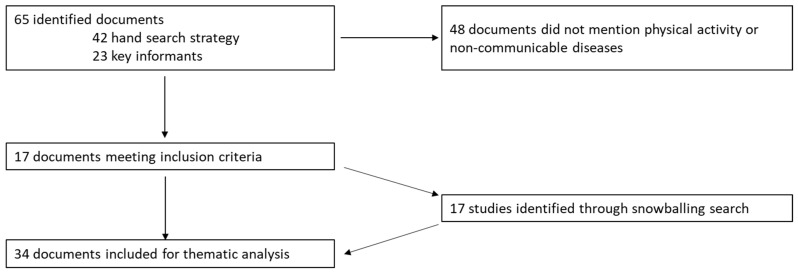
PRISMA flow diagram.

The hand search strategy yielded 42 policy documents (Figure 1). In tandem, key informants with proven expertise in the global physical activity research field were approached to identify additional policies relevant to this research. This yielded an additional 23 policy documents. The total yield of 65 documents was reviewed by two members of the research team (A.B.P. and A.W.). M.S.K., a senior team member, resolved any screening conflicts as they arose until consensus was achieved. Inclusion and exclusion criteria aligned to the GDAR protocol [25]. Documents were included if the policies made explicit or implicit reference to PA and had been published by an organization with a global lens in the years from 2000 to 2019. The exclusion criteria were as follows: a document did not make any reference to PA; a document was authored by an association that does not offer a global frame of reference position or policy agenda; a document was country- or region-specific; a document comprised resources that were designed primarily to guide program implementation or best practice (technical notes, best practices, and specific standards), status reports, or policy briefs. After screening, 48 documents were excluded, and 17 documents were selected for further analysis.

Subsequently, using the snowballing technique, the reference lists from included documents were scanned to identify additional documents. This process yielded 17 additional policies, adhering to the inclusion criteria, resulting in a total of 34 policies eligible for further qualitative analysis.

### 2.3. Data Analysis

Policies were uploaded and analyzed using a qualitative data analysis software—NVivo 12 [26]. An a priori codebook (Appendix A) was developed in line with research objectives, and thereafter an inductive thematic approach was used to analyze policy contents and a narrative analysis applied to understand the global trends in PA policies published between 2000 and 2019.

To understand the events underpinning the publication, agenda, or contents of the retrieved policies, we searched extracted policies for any references, background literature on national PA policy documents, or commentary on PA conferences, and approached experts in the field.

### 2.4. Policy Analysis Framework

The policy document analysis was guided by the Walt and Gilson policy triangle analysis framework [20]. The framework identifies four dimensions for consideration in policy analysis—context, content, process, and actors [20]. Keeping in line with our research objectives as well as available information, this policy review predominantly focused on the content dimension, and in particular, the evolution of this dimension, aligned to certain sentinel events and unfolding global dialogue. Content analysis pertained to the key discussions and recommendations made towards promoting population-wide PA.

## 3. Results

### 3.1. Policy Context: Global Physical Activity Policy Timeline and Sentinel Moments

**Figure 2:**
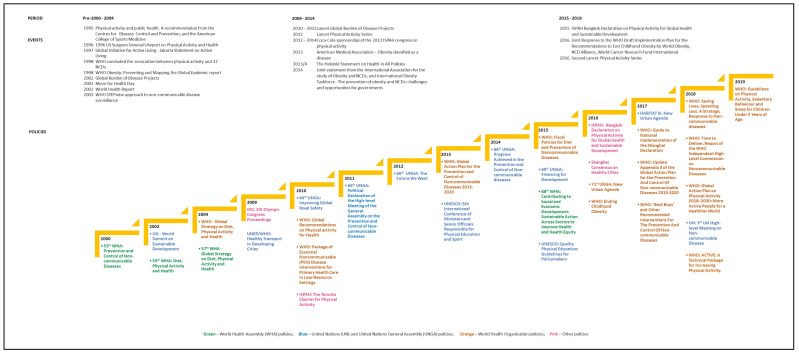
Global physical activity policies timeline.

We analyzed 34 global policies focused on PA, published between 2000 and 2019 [11,12,13,17,18,19,27,28,29,30,31,32,33,34,35,36,37,38,39,40,41,42,43,44,45,46,47,48,49,50,51,52,53,54]. Figure 2 illustrates the name, type, and timeline reflecting the approximate date of release of policies published during this time period. We further investigated events, occurring at a global scale, that may have been associated with, and may have preceded or coincided with publication of the identified policies (Appendix B).

The period between 2000 and 2019 saw an overall increase in the number of global PA policy publications, with three growth spurts occurring between 2002 and 2004, between 2009 and 2014, and between 2015-2019. The growth spurt aligned with the changes in the global understanding of the relationship between PA and NCDs, and more recently, sustainable development, social and environmental justice, and other co-benefits.

#### 3.1.1. First Growth Spurt: 2000–2004 Growth

In response to a sustained increase in global NCD morbidity and mortality, the 2000 World Health Assembly (WHA) through Resolution 53.17 called for the development of a global policy on the prevention and control of NCDs (Figure 2) [34]. This action culminated in the publication of WHO’s first policy, the WHO Global Strategy on Diet, Physical Activity and Health [25], focused on leveraging diet and PA as the main target for behavioral NCD interventions.

The promotion of PA as an NCD prevention strategy was based on evidence collated in prior years of 1995–1998, at both the national and global levels [55,56,57,58,59,60,61,62]. In 1995, the American College of Sports Medicine (ACSM) and the Centers for Disease Control and Prevention (CDC) published the first (USA) national guidelines on PA and public health, explicitly stating the health benefits linked to PA, particularly overweight and obesity prevention [55]. A later report—the 1996 United States Surgeon General Report—reiterated earlier sentiments by promoting PA in the prevention of other leading NCDs, including coronary heart disease, colon cancer, and anxiety and other depressive orders [56]. At the global stage, PA promotion was through the launch of WHO’s Global Initiative for Active Living [57] and publication of the WHO Obesity: Preventing and Mapping the Global Epidemic [58], which recommended increased PA for obesity prevention and control [58].

At this time, most of the evidence on PA and NCDs was derived from research in HIC with limited consideration of PA in LMIC settings [62]. Limited LMIC evidence was attributed to inadequate surveillance of PA behavior and health data management systems in most LMIC [62]. To address this, the WHO, launched a package of NCD surveillance instruments in 2003—the STEPwise (Stepwise Surveillance) tool—which provided a cost-effective standardized tool for capturing NCD behavioral risk factors across different contexts [62]. STEPwise remains pivotal in the reporting of the global burden of physical inactivity in relation to NCD occurrence [62].

Our search did not yield any policies published between 2005 and 2008.

#### 3.1.2. Second Growth Spurt: 2009–2014

In 2010, the WHO published its first stand-alone PA policy [26]. In tandem, PA was a key topic for deliberation at two United Nations General Assemblies (UNGAs)—2010–2011, thereby highlighting its growing relevance outside of the health sector [40,53]. PA-centric policies also started to emerge from other sectors and organizations during this period [29,44,48]. These policies emphasized the importance of strategies for increasing PA within the education, sport and recreation, transport, and urban development sectors. Publication of PA policies by sectors outside of health (Olympic Committee, United Nations Environment Program, United Nations Educational, Scientific and Cultural Organization) was potentially linked to the 2011 Rio Political Declaration on Social Determinants of Health [63], which enunciated the contributing role of other sectors in NCD etiology.

In 2010, the International Society for Physical Activity and Health (ISPAH) published the Toronto Charter for Physical Activity [47]. The charter, co-signed by actors from different sectors including academic institutions, advocated for countries to prioritize PA in the context of overall health and wellbeing, as well as economic and environmental sustainability [64,65]. Following the of the Toronto Charter’s publication, the gravity of the global physical inactivity prevalence was illuminated in the Lancet’s Physical Activity Series that labelled physical inactivity as a pandemic requiring global action.

The global advances in PA promotion during this period were not without controversy. It is suggested [66,67] that the popularity of PA for prevention of obesity (and, in turn, NCDs) was partly fueled by lobbying from the food production industry. Of note, it is alleged that in the period leading up to signing of ISPAH’s Toronto Charter, there was an increased activity by Coca-Cola (a food production company) to promote PA as a more viable NCD intervention target compared to diet [67,68]. One such incident was the controversial sponsorship of the fifth ISPAH conference by the Coca-Cola company between 2012 and 2014 [67]. It is suggested that in a bid to divert attention from diet as a key risk factor for NCDs, the company sponsored research that emphasized PA as a more important determinant of obesity and NCDs at the expense of dietary interventions [67]. In addition to sponsorship of existing events, other strategies included the formation and sponsorship of the Global Energy Balance Network, as well as support for the global “Exercise as Medicine” initiative [69,70,71].

#### 3.1.3. Third Growth Spurt: 2015–2019

On average, more global PA policies (average of four) were published per year in the period between 2015 and 2019 relative to previous years. The majority (*n* = 11/18) of the policies were published by the WHO, and the rest by finance, education, and urban development agencies (Figure 2) [11,13,19,27,28,30,32,33,37,38,43,44,45,49,50,51,52,54]. Compared to prior years, there was a shift away from mainly collating evidence on the link between PA and health, towards also outlining global PA action plans as well as integrating PA considerations into other policies. For instance, in 2016, the ISPAH Bangkok Declaration highlighted that PA promotion could positively contribute towards attainment of six 2030 Sustainable Development Goals (SDGs) (SDG3—Ensure healthy lives and promote wellbeing; SDG4—Quality education; SDG5—Gender equity; SDG11—Inclusive, safe, resilient, and sustainable cities and communities; SDG13—Climate change; SDG15—Life on land) [13,72,73]. Additionally, the ISPAH, with support from Thai Health Promotion Foundation (an autonomous government agency focused on empowering and coordinating individual and multisectoral health promotive activities in Thailand) [74] shaped the broader agenda of the ISPAH Bangkok Congress to underscore the need for multisectoral PA policy action in improving PA indicators [13,75].

In 2019, the WHO launched the first sub-population specific guidelines for PA targeting children below 5 years of age [52]. This potentially signified the continued discussion around the need to tailor various policies for specific groups, according to age, co-morbidities, and abilities. Prior to this point, although recommendations had been made for people across all age groups, there had been limited focus on sub-groups.

### 3.2. Policy Content

We identified three overarching themes in the reviewed policies. The first of these was a well-articulated global governance structure characterized by interdependent relationships between the perceived central global health agency (the WHO), national governments, and civil society. The second was the heightened attention towards structural factors contributing to physical inactivity and the need to address these using intersectoral approaches. The third was a dearth of PA policy recommendations that specifically considered the needs of women and adolescents.

#### 3.2.1. Key Global Physical Activity Policy Stakeholders

**Figure 3:**
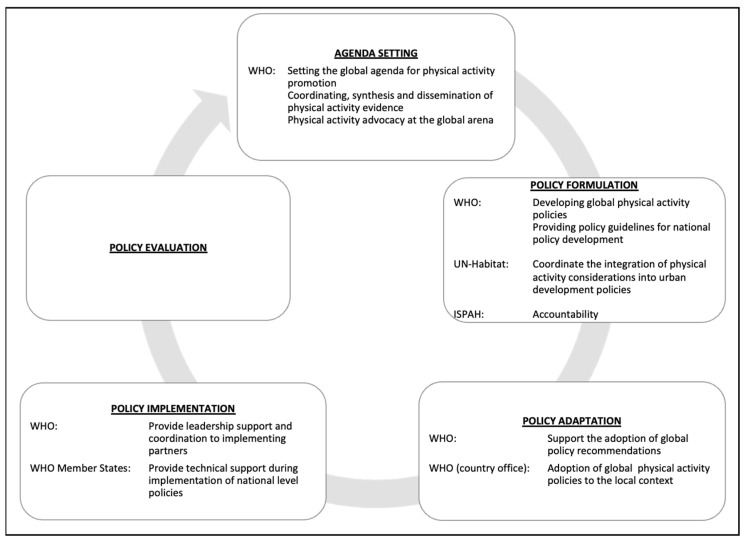
Highlighted stakeholder roles in the global physical activity policy development cycle. The figure indicates the five stages of policy development (Agenda Setting, Policy Formulation, Policy Adoption, Policy Implementation, and Policy Evaluation), stakeholders involved, and their perceived roles as indicated in reviewed policies. Abbreviations are as follows: WHO—World Health Organization, UN-Habitat—United National Human Settlements Programme., ISPAH- International Society for Physical Activity and Health.

Reviewed policies indicated a centralized global health governance structure. The WHO occupies a central and principal role, guides the global PA policy development cycle (agenda setting, formulation, adoption, implementation, and evaluation), and coordinates the involvement of other actors (non-state international agencies, national governments, and civil society) (Figure 3) [13,17,33,34,35,36,41].

- World Health Organization
  The WHO leads the global PA policy development cycle via three main avenues: providing leadership and coordination, evidence synthesis and dissemination, and PA advocacy (Figure 3). Leadership by the WHO, as described in the 53rd WHA, is important for centralizing all efforts to improve global PA [34]. To this effect, the WHO has a dedicated high-level commission, which was established in 2017 to tackle the growing NCD problem, with a sub-focus on reducing the prevalence of key risk factors, notably unhealthy dietary practices and physical inactivity. Through this commission and in partnership with global and national, state, and non-state actors, the WHO coordinates evidence synthesis (to establish the importance and viability of addressing physical inactivity as a global health indicator and target), develops relevant policies, and guides global advocacy for PA [11,17,34,36,41]. To realize its global PA mandate, the WHO relies on strategic partnerships with other UN agencies, international associations, member state governments, research institutions, and academia [34].

“WHO, in cooperation with other organizations of the United Nations system, will provide the leadership, evidence-based recommendations and advocacy for international action to improve dietary practices and increase physical activity, in keeping with the guiding principles and specific recommendations contained in the Global Strategy.” 2004 57th World Health Assembly-Global strategy on diet, physical activity and health.

- WHO Member states
  WHO member state governments were identified as the key implementing agents of global PA policies [13,17,28,33,35,36]. There was agreement, across policies, that success of the global PA initiatives was reliant on the successful implementation of policies at the national level, in order to create a global synergy.

“The role of government is crucial in achieving lasting change in public health. Governments have a primary steering and stewardship role in initiating and developing the Strategy, ensuring that it is implemented and monitoring its impact in the long term”—2004 57th World Health Assembly-Global strategy on diet, physical activity and health.

Realizing global PA policies at the national level was described as a three-step process: adoption and adaptation, implementation, and monitoring and evaluation. National governments, with guidance from the WHO’s country offices, were encouraged to firstly develop contextual national NCD prevention policies on the basis of recommendation from global policies [32,33,50], and thereafter pilot and implement these policies with assistance from the WHO and other non-state actors. Identified facilitators to implementation were political will, leadership will, and financial resources [18,31,50].

Financial resource allocation is of particular concern for LMICs, wherein national health budgets are often constrained with limited political or administrative will to allocate additional financial resources [32,50]. Furthermore, physical inactivity may not be of high priority in comparison to other health concerns, owing to insufficient local evidence and comparatively low physical inactivity prevalence in LMICs relative to HICs. Although these LMIC constraints are noted as concerns by WHO and UN policies [11,18,28,31,50], these policies did not provide actionable suggestions on how to address such challenges. Their focus tended to highlight the need to champion the “PA for NCD prevention” agenda across all facets of the government and garner support for its importance, thereby making it a key consideration in national and ministerial budgets.

In tandem with policy development and implementation, national governments were also tasked with setting up and maintaining monitoring, evaluation, and reporting mechanisms with support from global health agencies [11,12]. Such systems would allow for tracking of PA trends as well as providing evidence and learning insights for policy implementation. Additionally, surveillance systems were highlighted as important for holding national agents accountable for delivering on their mandates.

- Civil Society and Community Engagement

Civil society involvement in policy development and implementation was merged with actions from other actors including national and local governments, professional bodies, and philanthropic organizations [11,17,27,36]. Although the term civil society was mentioned in the four policies, there was no consistency in use of the term. For instance, 2016 ISPAH Bangkok Declaration and 2018 UN Time to Deliver suggests civil society as being separate from non-governmental organizations or global health initiatives.

Civil society is responsible for leading and promoting diet and PA initiatives within relevant state and non-state organizations whilst holding government agencies accountable for equitable interventions and empowering communities to voice their needs [11,17,28,38]. However, guidelines on the enactment of these roles were vague and limited within the national and sub-national levels.

“Civil society and nongovernment organizations have a central role in leading advocacy and monitoring accountability” 2018 World Health Organization-Global Action Plan for Physical Activity 2018–2030.

#### 3.2.2. Structural Determinants and the Role of Intersectoral Approaches to Address Global Physical Activity

Within WHO member state governments, ministries of health were designated to lead the adoption of global polices and implement all five stages of policy development. The push for national ministries of health to champion health in other sectors points to the need for intersectoral action to address physical inactivity across all levels and the commensurate governance structures to promote this [13,19,49]. Proponents of intersectoral collaboration advocated that the inclusion of collaborative PA considerations across policies from multiple sectors would result in synergized responses and outcomes.

“The actions needed to increase physical activity require multi-sector leadership, partnerships, and sustained commitment as well as targeted allocation of resources”—2016 ISPAH-Bangkok Declaration on Physical Activity for Global Health and Sustainable Development.

National ministries of health were firstly tasked with raising awareness on the linkages between physical inactivity and NCD occurrence, economic activity, and social inequality; mapping out action plans on how other sectors could contribute to PA initiatives [17,35], advising on the integration of PA concerns in polices from other sectors, and coordinating and facilitating intersectoral collaboration towards reducing PA at the national and subnational levels

Outside of health, other key sectors identified for direct policy implementation were education [11,12,13,17,18,30,31,32,36,37,44,46], transport and urban planning [11,12,13,18,19,28,31,32,36,49,50], finance, and political leadership [11,12,18,19,38,49]. Other noted sectors included the sports and leisure industry, local government, and community organizations. The broader contributing roles for the education, transport, and urban planning sectors were well outlined in the WHO’s 2018 Global Action Plan on PA and similar policies, but there was a paucity of information on exactly how other sectors such as trade and commerce, energy, social welfare, energy, and humanitarian relief might directly contribute towards improving population PA.

“Action by sectors other than health, at the national, regional and local level can substantially contribute to improved health and health equity, for example, through policies involving social protection, food security, education, poverty reduction, transportation, environment, finance, trade and commerce, and taxation and legislation on the marketing of certain products. Some sectors work more closely with the health sector than others, depending on two key factors: common interests and co-benefits.”—2015 68th World Health Assembly-Contributing to social and economic development: sustainable action across sectors to improve health and health equity.

#### 3.2.3. Policy Focus on Women and Adolescents

To complement the systemic recommendations, the WHO set out PA recommendations at the individual level in six policies [12,17,18,31,35,36]. These recommendations have since been consolidated through publication of the 2020 WHO Guidelines on Physical Activity and Sedentary Behaviour. Between 2000 and 2019, recommendations set out for individuals were broadly grouped according to age groups: for children and young people (5–17 years), adults (18–65 years), and older persons (greater than 65 years). Adults and the older adults were recommended to engage in at least 150 min of moderate PA or 75 min of vigorous PA each week. It was also suggested that both groups should engage in muscle strengthening exercises on at least 2 days of each week. Additional exercise above the minimum was also advised for additional health benefits. Sufficient PA requirements were higher for the children and young people ages 5–17, with a recommendation of at least 60 min of daily PA, and muscle and bone strengthening exercises at least three times per week to gain health benefits.

Of particular interest to this research study, five policies [13,18,31,32,50] made specific references to the urgent need for strategies to improve PA in adolescent age groups (includes children and the youth) and addressed PA in all aspects of their daily lives including education and transportation. This is highlighted by the presence of adolescent specific PA guidelines (60 min of daily moderate to vigorous intensity PA) for health and natural development benefits; spotlighting adolescent-adjacent activities (play, games, sports, transportation, recreation, physical education, or planned exercise, in the context of family, school, and community activities); and promotion of adolescent physical literacy by incorporating physical education into basic education curricula. However, context-specific recommendations were not stipulated.

An acknowledgement of the gender bias in PA participation was made across five policies [12,13,36,49,53]. These policies indicated that women and girls are less likely to engage in PA compared to men and as such were at more risk for PA-related NCDs. Reduced participation in PA was attributed to factors that included an inordinate burden for child caregiving resulting in time constraints, as well as limited access to safe and affordable opportunities for women to engage in PA.

“Policy actions which promote physical activity through improved access to safe and affordable opportunities to participate in sport and physical activity by girls and women, particularly those in marginalised and disadvantaged communities, can contribute to ending discrimination (Target 5.1) in sports and physical activity as well as contribute to the prevention of NCDs and ensure healthy lives and promote wellbeing”—2016 ISPAH-Bangkok Declaration on Physical Activity for Global Health and Sustainable Development.

## 4. Discussion

This study identified 34 global PA policies published between 2000 and 2019, discussed sentinel moments surrounding the publication of policies and analyzed policy contents. Overall, shifts in global PA policy perceptions mirrored the increased appreciation for nuanced prevention strategies to reduce worldwide NCD incidence and mortality. Policies described the global PA governance structure of which the WHO steered the global agenda, whilst its member states were responsible for policy implementation. In line with the push towards intersectoral action, policies alluded to the need for increased input from other sectors to collectively address the systemic determinants of physical inactivity. Lastly, we noted that there were minimal considerations and recommendations for sub-population groups, including women and girls, as well as children and adolescents.

The period between 2000 and 2019 saw an overall increase in the number of global PA policy publications with three growth spurts occurring between 2002 and 2004, between 2009 and 2014, and between 2015-2019. Research evidence and advocacy by various actors collectively influenced the focus and nature of global PA policies over time. Health research evidence initially cemented the link between physical inactivity and the increasing non-communicable disease incidence (2002–2004) and later turned the spotlight on underlying economic, socio-cultural, and structural determinants fueling the physical inactivity pandemic (2009–2014). Complementary civil society organizations, notably ISPAH [13], through advocacy efforts were influential in framing the PA discussion as being important not only for NCD prevention and control, but also highlighting how PA considerations could directly contribute towards attainment of the UN’s 2030 SDGs. The impact of these influences can be seen in the post-2010 increase in stand-alone PA policies, as well as a PA focus in policies from organizations outside of the traditional health sector.

Publication of standalone or PA-adjacent polices from different sectors points towards information sharing partnerships between different actors, notably health agencies, academia, and civil society. Such partnerships were highlighted as beneficial to the promotion of PA across different spheres. However, the nature of partnerships should be scrutinized. This is considering the controversial partnership between PA research and advocacy actors and a food production cooperation—Coca Cola [66,67,68]. This was through the sponsorship of the 2012 ISPAH conference by Coca-Cola, as well as funding for research promoting the “Exercise is Medicine” rhetoric [66,67,68]. This promotion was to place more emphasis on PA intervention as a best buy for NCD preventions relative to diet. There was criticism of Coca-Cola sponsorship at the time, and this has contributed to discussions, within the global health arena, on the conflicts of interest posed by the participation in PA research and advocacy of large corporations, as these corporations often contribute to obesogenic environments [66,71].

Three key actors—the WHO, national governments, and civil society—were identified. The first global PA policy published between 2000 and 2019 was from the WHO, and subsequently half of the policies analyzed in this study are publications from this organization. Furthermore, in 2017, the WHO established a dedicated Thematic Working Group on Physical Activity. This is of particular importance to the global physical inactivity agenda because, historically, when the WHO takes note of a particular health-related issue it inevitably changes its landscape. For example, in response to the smoking pandemic, the WHO in 2003 adopted the WHO Framework Convention on Tobacco Control [76], which fostered the adoption and implementation of tighter regulation of the tobacco industry among WHO member states [76,77]. Whilst the contribution of this treaty with respect to smoking rates is contentious, it was instrumental in shaping the global narrative on the regulation of the tobacco industry, as evidenced by the adoption of these regulations in some WHO member state national policies [76,77]. Spotlighting of PA by the WHO is of importance because even though PA’s importance for NCD prevention and control is well acknowledged, population PA interventions are often less prioritized (both globally and nationally) compared to the other three top NCD risk factors—unhealthy diets, tobacco use, and harmful use of alcohol. Therefore, an emphasis on PA by the WHO along with advocacy by civil society actors will advance the global PA agenda.

National governments were identified as the main stakeholder steering the implementation of the global PA agenda at the local context. This was through establishment of national PA polices (in line with the global agenda), facilitating integration of PA principles in sectoral policies, and PA promotion to the public. A recent study examining the presence of PA policies in Cameroon encouragingly found 17 PA-relevant policies published across different sectors [78]. However, across the policies, there was almost no explicit link between PA and NCDs [78]. At the global scale, a recent study found that across 76 countries, 92% of countries had at least one formal PA policy document predominantly published by the sport, education, health, and recreation and leisure sectors [79]. On a larger scale, the Global Observatory of Physical Activity published two Physical Activity Almanacs (PAA) in 2016 and 2021 [80,81], using rigorous methodology for data collection to report on the existence of stand-alone national PA plans. In 2013, 139 countries were represented in the PAA, and of these, 26.6% reportedly had a stand-alone national PA plan [80]. However, none of the represented countries in the Africa region (AFRO) (*n* = 14), in the Eastern Mediterranean (EMRO) (*n* = 12), and in South-East Asia (SEARO) (*n* = 9) had a stand-alone national plan for PA [80]. In the 2021 report, 217 countries were included, and of these, only 18% had a stand-alone plan for PA, but an additional 37% included PA as part of a national NCD plan or policy [81] and 17, 14, and 12% of countries in the AFRO (*n* = 47), EMRO (*n* = 22), and SEARO (*n* = 11) regions, respectively, now have national PA plans [81]. This shows a remarkable trend toward greater national PA policy engagement in previously under-represented areas of the world. However, the actual implementation of these policies lags.

The gap between adoption and implementation of PA policies has been attributed to a number of reasons such as limited financial and human resources (across sectors), competing priority public health needs within the health sector, and fragmented intersectoral action [82]. Further to this, global PA policy recommendations and interventions such as infrastructural development to encourage non-motorized transport and population health education initiatives require resources that may be scarce to begin with in some LMICs. This juxtaposition between policy adoption and implementation highlights the need for greater consideration on ways to improve PA indicators in the absence of additional and equitable distribution of resources. In essence, without any increases to national health budgets or new infrastructural projects, what strategies can be employed now to improve population PA? At present, there is a dearth of knowledge of such interventions in LMICs, but increasingly more studies are being conducted to this end. A recent review [83] identified examples of implemented community awareness campaigns; community and social support strategies; and school-based and infrastructural interventions conducted in 15 countries, including Iran, [84] South Africa, [85,86] Brazil [87], China [88] Pakistan [89], and Colombia [90].

There was a concerted effort to promote intersectoral approaches for PA initiatives across policies. This was driven by research and industrial evidence demonstrating that synergy from intersectoral efforts has a greater impact in improving population PA. Although the need for broader intersectoral action was relayed, there was limited guidance on how each sector can practically contribute towards suggested recommendations. The roles of the health, education, transport, and urban planning sectors are well articulated. However, for sectors such as energy, trade, social welfare, and the private sector, there are no clear entry points for intersectoral action to improve PA, nor is there guidance on how these sectors can integrate PA considerations in their own policies. The absence of clear and distinct roles at the global level is mirrored in national level PA policies. Evidence from a study exploring Cameroon’s national PA policies found that PA policies were lacking in some key sectors such as the transport sector [78]. This is an important area in which international, national, and local health agencies should provide more guidance, particularly for LMICs undergoing economical and infrastructural development, whereby clear intersectoral governance and reporting structures could facilitate cooperation among sectors. The subsequent adoption of intersectoral PA policies would ensure that future cities are developed to accommodate and promote active living. Furthermore, in considering the engagement of other sectors outside of health, traditional metrics for PA surveillance may need to be expanded to accommodate these sectors. For instance, the 2018 Global Action Plan for Physical Activity 2018–2030 lists the creation of active environments as an objective for improving population PA [11]. To realize this objective, the built infrastructure and urban planning sectors need to incorporate features that encourage PA. Ergo, to track progress on this objective, PA surveillance metrics would need to incorporate indicators that capture the impact of improved urban design on population PA.

With much focus placed on defining governance structures and encouraging systemic interventions, there was limited discussion, across all policies, on the sub-population dynamics of PA. Specifically, disparities in PA amongst sub-populations that face peculiar challenges including women and girls, children and adolescents, people living in low socio-economic environments, and those living in conflict regions are documented in literature. In the case of women and girls, five policies [12,13,36,49,53] highlighted the gendered dimension of PA, with women and girls less likely to engage in PA, in part, due to limited availability of safe and affordable facilities. Therefore, to ensure equitable access and advances to PA, there is a need for increased global sensitization on the lived realities of these vulnerable populations to bolster the global discussion towards more context relevant interventions. This could be done through publication of policies focused on specific sub-population groups by leading health agencies, such as WHO’s Framework for the Implementation of the Global Action Plan on Physical Activity 2018–2030 in the WHO African Region [91], and the recently published WHO Guidelines on physical activity, sedentary behavior and sleep for children under 5 years of age [52]. Similar guidelines can be published for other population subgroups to provide much needed guidance for national governments.

## 5. Conclusions

The last two decades have brought about a commendable increase in the number of policies centered on addressing global physical inactivity. Initially rooted in NCD prevention and control, or linked to diet for the prevention of obesity, the conversation has broadened to understand the key systemic factors influencing present day PA and the subsequent need for intersectoral action to address them. In the 34 policies reviewed in this study, we noted that PA governance was hierarchical, with WHO leading global initiatives and national governments adopting and implementing international policies. As more countries develop and implement PA polices, more attention should be placed on strategies to report and evaluate the implementation on policy actions. Intersectoral collaboration is highlighted as an action point going forward; however, outside of the health, education, transport, and urban planning sectors, the definitive contributions for other sectors remain vague. To this end, it is imperative for both global and national health agencies to expressly engage with representatives from other sectors and collaboratively map out potential avenues in which they can integrate the promotion of PA within internal and intersectoral policies. There was little articulation of the socio-cultural and built environment barriers negatively impacting PA among sub-population groups, including women and girls. Going forward, at the global scale, these barriers should be more clearly acknowledged, so as to improve understanding amongst relevant stakeholders and foster the development of targeted and contextually relevant policies to guide sub-population PA interventions.

## Data Availability

Data sharing is not applicable to this article. No new data were created or analyzed in this study.

## Appendix A

**Table A1:** Codebook themes.

Name	Description
Adolescents	Specific references to youth or adolescents are made
NCD diet	If adolescents are referenced in relation to NCD and diet
NCD diet physical activity	If adolescents are referenced in relation to NCD, diet, and physical activity
NCD only	If adolescents are referenced in relation to NCDs only
NCD physical activity	If adolescents are referenced in relation to NCD and physical activity
NMT physical activity	If adolescents are referenced in relation to non-motorized transport and physical activity
Gender	Specific references to gender are made
NCD diet	If gender is referenced in relation to NCD and diet
NCD diet physical activity	If gender is referenced in relation to NCD, diet, and physical activity
NCD only	If gender is referenced in relation to NCDs only
NCD physical activity	If gender is referenced in relation to NCD and physical activity
NMT physical activity	If gender is referenced in relation to non-motorized transport and physical activity
Global policy reference	Specific reference to global policy intentions, such as WHO Best Buys
Governance	
Accountability	Are there any suggestions regarding accountability? What are the accountability mechanisms for the countries assigned as responsible?
Leadership	Who has been identified as leaders for implementation?
Level of commitments	Reference to regional or international commitments?
Responsibility	Governance—who should coordinate a response?
Sectors	Which sectors are most important?
Implementation	Accompanying implementation plans and related timeframes
Challenges	E.g., what does the document say are challenges for implementing this strategy/policy?
Needs	What is needed regarding implementation?
Intersectoral	Specific mentions or encouragement of involvement of more than one sector
Ministry—Agency	Primary ministry/agency (e.g., WHO) responsible
Other	Other ministries named
NCD Action—Who	Who should take action on NCDs? (specific proposals)
NCD Problem—Why	Why are NCDs a problem? (How is this framed in the policy document?)
Cost	NCDs are a problem because they hamper countries’ economic growth and affect their finances
Mental health	
Mortality and morbidity	NCDs are a problem because they contribute to the burden of mortality, morbidity, and disability
Socio-economic inequalities	NCDs are a problem because they contribute to social and economic inequalities.
Other	Any other key/interesting aspects of relevance that we may not want to include in this study, but that might be worth noting
Physical activity NCD cause	Framing and beliefs specific to physical activity: what are the causes of physical activity-related NCDs? Beliefs about the problem
Physical activity proposal	Framing and beliefs specific to physical activity
Data collection and monitoring	
Education	Policy content specific to physical activity (What should be done?): physical activity education
Environment	Framing and beliefs specific to physical activity—specific proposals about the environment/air pollution
Health	Policy content—specific to physical activity (What should be done?): health systems
Promotion	Policy content specific to physical activity (What should be done?): social marketing and health promotion campaigns
Transport	Framing and beliefs specific to physical activity: specific proposals about transport
Urban planning	Framing and beliefs specific to physical activity: specific proposals about urban planning (including housing)
Purpose	Stated purpose of the document (in particular, note if a focus on NCD and, where present, if there is a focus on diet and physical activity)
NCD diet	If the purpose has a focus on NCD and diet
NCD diet physical activity	If the purpose has a focus on NCD, diet, and physical activity
NCD only	If the purpose has an NCD focus only
NCD physical activity	If the purpose has a focus on NCD and physical activity
NMT physical activity	If the purpose has a focus on physical activity tackled as part of non-motorized transport policy
Resource	Resourcing—both diet and physical activity—what commitments regarding resourcing are being made?
Source	Where should the money come from?
Target	Target population(s) (How is this construed?)

## Appendix B

**Table A2:** Global events coinciding with the publication of global physical activity policies.

Year	Event	Description
**Pre-2000–2004**		
1995	Physical activity and public health; a recommendation from the (CDC) and the (ACSM) [55]	On the basis of epidemiological evidence published pre-1995, the CDC and ACSM (leading public health agencies in the USA), for the first time, released a joint message recommending that adults should engage in at least 30 minutes of physical activity on most days of the week as a health promotion and disease prevention measure.
1996	1996 U.S. Surgeon General’s Report on Physical Activity and Health [56]	This report assessed the role of physical activity in NCD prevention. It was instrumental in highlighting that the benefits of physical activity engagement were extensive to include risk reduction for major NCDs, symptom alleviation for some mental health conditions, and weight control.
1997	GIAL—Jakarta Statement on Active Living [57]	The WHO, through the GIAL, instituted a physical activity health promotion drive based on research evidence, stating that daily physical activity enhanced overall health and that increases in sedentary lifestyles reduced opportunities for the population to engage in physical activity.
1998	The WHO concluded the association between physical activity and 17 NCD [86]	This viewpoint, published by a leading global health organization, advocated for physical activity as an important lever in the fight against increasing global incidence of NCDs.
1998	The WHO published the Obesity: Preventing and Mapping the Global Epidemic report [92]	Along with diet, the WHO identified physical activity as an amenable target reducing the worldwide incidence of obesity and subsequently NCDs.
2002	Global Burden of Disease Projects [59]	In 2002, the WHO updated findings from the World Bank-commissioned 1990 Global Burden of Disease Study. NCDs were posted as a major concern.
2002	Move for Health Day [60]	Campaign by the WHO to emphasize the importance of fitness and promote physical activity and healthy behaviours.
2002	World Health Report [61]	This report listed physical inactivity as one of the main risk factors contributing to global NCD morbidity and mortality.
2003	STEPWise [62]	The WHO launched a globally standardized data collection tool—STEPS—to report on NCD behavioural risk factors (including physical activity) trends across different contexts, particularly LMIC.
**2009–2014**		
2010–2012	Lancet Global Burden of Disease Projects [93]	This report highlighted the continued increase in global NCD morbidity and mortality, as well as the growing double burden of disease challenge in LMIC contexts. This provided further evidence on the need for feasible interventions to improve health outcomes.
2012	Lancet Physical Activity Series [6,94]	Publication of this series was significant as this was the first series by a leading journal dedicated to addressing physical activity as a pandemic.This series further enunciated links between physical inactivity and health, and advocated for crosscutting interventions across all societal (micro, meso, and macro levels).
2012–2014	Coca-Cola sponsorship of the 2012 ISPAH congress on physical activity [63,64,72]	Coca-Cola—one of the leading producers of sugar-sweetened beverages—sponsored the 2012 ISPAH conference, which was focused on physical activity research and practices. This triggered global conversations on the ethical implications of large cooperation’s involvement in physical activity initiatives given their well-articulated contribution towards unhealthy behaviours.
2013	AMA—Obesity classified as a disease [95]	For the first time, obesity was recognized as a disease as opposed to being a condition by AMA. This was important for bringing more attention towards the disease and therefore more concerted efforts towards prevention and treatment. Diet and physical activity were highlighted as key areas for intervention.
2013/4	The Helsinki Statement on HAIP [96]	The HIAP approach advocated for the inclusion of health considerations across sectoral policies. This brought to the fore the need for more health behaviour considerations in other sectors, such as transport and urban planning, that directly influence opportunities to engage in physical activity.
2014	International Association for the study of Obesity: International Obesity Taskforce—The prevention of obesity and NCDs: challenges and opportunities for governments [97]	Key global consortiums focused on obesity highlighted physical activity as an important obesity and NCD prevention strategy and urged national governments to take action through a series of recommendations.
**2015–2019**		
2015	ISPAH Bangkok Declaration on Physical Activity for Global Health and Sustainable Development [13]	ISPAH-linked physical activity to eight SDGs. This declaration framed physical activity in the context of economic development.
2016	World Obesity, NCD Alliance, World Cancer Research Fund International—Joint Response to the WHO Draft Implementation Plan for the Recommendations to End Childhood Obesity [58]	This was a joint response that was also co-signed by 20 other organisations working on varying aspects of NCD prevention. This signified the power inter-organizational alliances in critiquing WHO policies on obesity prevention measures.
2016	Second Lancet Physical Activity Series [83]	As a precursor to the 2012 series on physical activity, this series provided an update on the state of global physical inactivity and associated health effects. In line with transdisciplinary interest, the series, for the first time, also provided a global estimate for economic burden of physical inactivity.
